# Assessment of pesticide use and pesticide residues in vegetables from two provinces in Central Vietnam

**DOI:** 10.1371/journal.pone.0269789

**Published:** 2022-06-13

**Authors:** Chau Nguyen Dang Giang, Dang Bao Chau Le, Van Hop Nguyen, Thai Long Hoang, Thi Van Thi Tran, Thi Phuong Linh Huynh, Thi Quynh Trang Nguyen

**Affiliations:** 1 Department of Chemistry, University of Sciences, Hue University, Hue City, Vietnam; 2 Department of Sociology, University of Sciences, Hue University, Hue City, Vietnam; 3 Sociology, Anthropology in Water Management, French National Research Institute for Sustainable Development (IRD) in Hanoi, Kim Ma, Ba Dinh, Hanoi, Vietnam; 4 Faculty of Environmental Sciences, Saigon University, District 5, Ho Chi Minh City, Vietnam; Tocklai Tea Research Institute, INDIA

## Abstract

Pesticide residue in food, especially in vegetables, is one of the important parameters to assess food safety. This study evaluates the pesticide use in vegetables from two provinces in Central Vietnamand and present data on pesticides detected in vegetables sampled from the sites. The potential health risk associated with the contamination of four commonly used pesticides in different vegetables is also discussed. Both household surveys and monitoring campaigns were conducted. The survey showed that improper pesticide application, storage, and waste disposal prevailed at the study sites. Only 20% of the respondent were aware of pesticide toxicity. As a result, pesticides were detected in 81% out of 290 vegetable samples collected at harvesting time. Up to 23% of samples had pesticide residues above the Maximum Residue Limit values. The highest total pesticide concentration quantified in vegetables in Thua Thien Hue was 11.9 mg/kg (green onions), and in Quang Binh was 38.6 mg/kg (mustard greens). Median residue levels of individual pesticides in vegetables ranged from 0.007 to 0.037 mg/kg. Among the ten target pesticides, cypermethrin, difenoconazole, and fenobucarb were detected at the highest frequencies (72%, 41%, and 37%, respectively). Pesticide residues varied between seasons at both study provinces. Pesticide contamination in the wet season was significantly higher than in the dry season. This study also discovered a potential health risk associated with fipronil residues in vegetables in Thua Thien Hue province. The paper provides recommendations for mitigation measures (both technological and social) in reducing potential health risks linked to pesticide use in vegetables in the region.

## Introduction

Pesticide usage in agricultural production is an issue that generates a great deal of heated debate. On the one hand, farmers have incessantly relied on pesticides for pest control, crop protection, and crop productivity support. On the other hand, pesticide residues in food cause harmful effects to human health. Since the organochlorine pesticides were banned from being used for crop protection worldwide [1], a wide range of pesticide classes has been introduced to the market such as carbamates, pyrethroids, phenyl pyrazoles, etc. The outstanding advantages of these later generation pesticides are their high acute toxicity to the targets, fast decomposition, and less bioaccumulation potential [2]. However, these merits accidentally form a general misconception among farmers that these pesticides are not toxic, do not pollute the environment, or do not affect human health. Consequently, it results in the misuse and abuse of pesticides, which then causes a risk of dietary intake of pesticides.

Plentiful studies worldwide have provided evidence related to the inadequate management and ignorance in the use of pesticides. A few highlights to note include overdosage [3, 4] lack of adequate personal protective equipment when handling pesticides, leading to exposure [5, 6]. Careless usage is in pair with low penetration of advanced farming practices (such as Integrated pest management (IPM), Good agricultural practices (GAP)) [7, 8], trading of fake pesticides, and shortcoming in management and distribution of pesticides [9]. Vietnam is not an exception. Knowing to be one of the leading countries in rice [10] and vegetables [11] production worldwide, the usage of pesticides is well documented in this country. Previous works have revealed the situation of pesticide use and management across Vietnam. The main findings of those studies [12–14] emphasize that pesticides are being used, stored, and disposed of improperly. These persisting problems generate potential health risks of pesticide exposure and adversely impacts on products’ values, thus hindering the sustainability of agricultural development as a whole.

Currently, from the human health viewpoint, monitoring pesticide residues in agricultural production is considered as the main measure to assess food safety [15]. The ubiquitous pesticide residues in foodstuff have been reported worldwide [16–20]. In these studies, most of the collected samples contained pesticides in varying concentrations. These publications also have provided alarming evidence of pesticide residues in vegetables and/or fruits exceeding Maximum residue levels (MRLs) allowed by either FAO and WHO (the Codex Maximum Residue Limits for Pesticides [15] or national MRLs. They provide insights into how the Estimated daily intake (EDI) exceeds the specified Acceptable daily intake (ADI) value. An additional concern is the existence of residues of multi-pesticides in the analyzed samples [21–23]. It is worth noting that the co-occurrence of pesticides might cause a synergistic effect that puts consumers at higher health risk [24].

In Vietnam, only a little information is available on pesticides in food samples. Hoai et al. [25] found residue levels of fenobucarb, trichlorfon, cyfluthrin, and cypermethrin in vegetable and tea samples collected in Hanoi that were higher than allowable (i.e. MRL) levels. Ngoc et al. [26] quantified pesticide residues in 350 vegetable and fruit samples including cabbage, broccoli, cucumber, and water spinach collected also in the Hanoi, and detected a concerning level of cis-permethrin, chlorpyrifos, and trans-permethrin. In Quang Binh province, a study by Nghiem in 2005 [27] reported that 169 out of 360 analyzed samples (47%) were contaminated by pesticides, including some banned pesticides such as gamma-benzene hexachloride (BHC), heptachlor epoxide, endosulfan I, methyl parathion, dichlorvos, and prothiofos. However, the research on the issue has been limited and scattered in this country.

Given the lack of available knowledge and the importance to provide more understanding of pesticide use and residues in vegetable production in Central Vietnam, this research seeks to: i) investigate the current status of pesticide use, ii) link it with pesticide residues in the main vegetables cultivated in North Central Vietnam, and iii) figure out if any potential health risk might occur when consuming vegetables from the study sites.

## Materials and methods

### Study sites

North Central Vietnam is a mountainous area, where the inhabited and cultivated land strip is squeezed between the mountainous upstream area and the coastal sand dunes. The area comprises six provinces (Thanh Hoa, Nghe An, Ha Tinh, Quang Binh, Quang Tri, Thua Thien Hue). The region has a typical tropical monsoon climate with high drought in the dry season (February to August) and humidity and heavy rainfalls in the wet season (September to January) [28]. Based on a desk survey and expert interviews, four communes in Thua Thien Hue and two communes in Quang Binh provinces, all with intensive vegetable production, were selected for this study. The specifications of the communes are described below:

In Thua Thien Hue, Quang Thanh commune in Quang Dien district was selected as representative for vegetable cultivation on lowlands. The commune has a total agricultural land of 684.79 ha, of which vegetable area accounts for 30.7 ha (4.5%). Huong An commune in Huong Tra district is located nearby a mountain area. The total agricultural area is ca. 579.5 ha, of which 53.5 ha (9.2%) is used for vegetable farming. Dien Hai commune in Phong Dien district represents the long tradition of vegetable production in the coastal lagoon area. The agricultural land area is ca. 544.9 ha with 42.4 ha (7.8%) used for vegetable farming. Quang Tho commune in Quang Dien district is located in the coastal plains. The commune has the largest area of pennywort (Centella Asiatica) in the province with a total area of about 42.1 ha (*Annual report from Department of Agriculture and Rural development*, *Thua Thien Hue province*, *2018*).

In Quang Binh, two communes of Le Thuy district were selected for field survey. The district has a total vegetable farm of ca. 41 ha and a great diversity of vegetable types. The first commune chosen is Cam Thuy, representing the vegetable production on sandy soil. The second commune is Hong Thuy, representing the largest vegetable cultivation area of the province, accounting for ca. 150 hectares. (*Annual report from Department of Agriculture and Rural development*, *Quang Binh province*, *2017)*

This study combined both household survey and monitoring study aiming to identify the linkage between pesticide use and the related residues in vegetables at the study sites.

### Household survey

To investigate the use of pesticides in vegetable production in Thua Thien Hue and Quang Binh provinces, both desk study and field survey were carried out. The survey was conducted from May to July 2018, using structured questionnaire (written form), with the approval of the Department of Natural Resources and Environment (DONRE) of Thua Thien Hue province and the DONRE of Quang Binh province.

From the beginning, four stratums (subpopulations) were defined for the survey: i) select a number of communes in the proposed district of each province; ii) select a number of villages in selected communes; iii) select household in selected villages; iv) select a member of the household to interview. The interviewed households were identified based on the suggestion of local authorities, on the consent to participate in the interview and the accessibility of households for interviews. In total, 233 households (155 from Thua Thien Hue and 78 from Quang Binh) were interviewed. Only households involved in vegetable farming were selected with a minimum cultivated area of above 500 m^2^.

The structured questionnaire developed and applied consisted of three parts. The first part explores the general demographics of the households and farm characteristics. The second part focuses on pesticide use and related farmers’ knowledge and attitudes. The last part defines food consumption and body weight (of respondent) for calculation of EDI. In addition, 11 farmers were randomly chosen from 233 households during the survey for in-depth interviews.

### Target pesticides and vegetables sampling

Selection criteria for pesticide residue monitoring were: i) commonly used pesticides by the local farmers as derived from the household survey, ii) synthetic chemical pesticides, and iii) pesticides measurable by GC-MS/MS instrumentation. As a result, ten priority pesticides were selected, namely the three herbicides acetochlor, fluazifop-p-butyl, and pretilachlor; the three insecticides cypermethrin, fenobucarb, and fipronil; and the four fungicides difenoconazole, isoprothiolane, tebuconazole, and trifloxystrobin. The general properties of studied pesticides are provided in the **S1 Table**.

Vegetables selected for this study are vegetables that are most frequently cultivated and consumed at the study sites based on the household survey result. Mustard greens (Brassica junceaf), lettuce (Lactuca sativa), green onions (Allium fistulosum), and pennywort (Centella asiatica (L.) Urb) were therefore selected for this study.

Sampling: In the 6 communes selected above, a total of 290 vegetable samples, i.e. 100 mustard green samples (50 in Thua Thien Hue, 50 in Quang Binh), 86 lettuce samples (48 in Thua Thien Hue, 38 in Quang Binh), 84 green onion samples (44 in Thua Thien Hue, 40 in Quang Binh), and 20 pennywort samples in Thua Thien Hue, were collected from November 2018 to June 2019. The samples were manually collected on the day of harvest. Half a kilogram of each sample (entire plant) was taken, wrapped in aluminum foil, and transported within 24 hours with ice-cooling to the laboratory (Hue University, Vietnam). The samples were then frozen at -20°C (to inhibit the degradation of the pesticides) prior to analysis.

### Chemicals and reagents

Pesticide standards, surrogate standard (δ-HCH), and internal standards (fluorene-D10, phenanthrene-D10) were sourced from Sigma Aldrich (purity > 97%, USA). Stock solutions (1000 μg mL-1) of the ten pesticides and the surrogate were prepared in acetone and stored at -20°C. Working solutions were prepared in toluene. Stock solutions of fluorene-D10 and phenanthrene-D10 (1000 μg mL-1) were prepared in toluene. All employed solvents were of HPLC grade, including n-hexane, acetone, ethyl acetate, and toluene (J.T. Baker, Deventer, The Netherlands). Sodium sulfate, glass fiber filters (47 mm, pore size 1.6 μm, Whatman, England) and ENVI-Florisil Supelclean (500 mg/3 mL) normal phase cartridges for solid-phase extraction were purchased from Sigma-Aldrich (USA), and activated carbon from Merck (Germany).

### Analytical procedure

The analysis method was based on our previous work as described in [29]. A detailed description of the protocols is provided in the **S1 Text**. Gas chromatography—triple quadrupole mass spectrometry system (GC-MSTQ model 8040, Shimadzu, Japan), employing an Rtx-CL pesticide capillary column (30 m x 0.25 mm, film thickness 0.25 μm, Restek, USA), was used for pesticide separation and detection. The triple quadrupole mass spectrometer was operated with multiple reaction monitoring (MRM) mode.

Method quality control was carried out following the SANTE/12682/2019 [30]: Linearity between detector signal and pesticide concentration was established with two sets of calibration curves, including solvent calibration and matrix-matched calibration (five levels 5, 10, 50, 100, 200 ng/mL), of which, the deviation of back calculated concentration from true concentration was also calculated. Spike recovery and repeatability—assessed via relative standard deviation (RSD) percent—of the analytical method was determined by conducting five replicates of blank samples (green onion and mustard green matrices) spiked with 5 ng/g, 20 ng/g, 100 ng/g, 500 ng/g and 2000 ng/g of the studied pesticides. The limit of detection (LOD) of each target pesticide was achieved by analyzing seven spiked samples of mustard green at a level of 10 ng/g, which was then calculated by LOD = 3.14 × SD (3.14 is the t value (one-tailed) for a 99% confidence level with six degrees of freedom, and SD is the standard deviation of seven replicates) [31]. The detailed quality control results are shown in the **S2 and S3 Tables**. Briefly, the spike recovery rates of the studied pesticides at 5 ng/g for both mustard greens and green onions varied in the range of 73% to 107%, at 20 ng/g were from 83% to 111%, at 100 ng/g were from 82% to 103%, at 500 ng/g were from 88% to 99%, and at 2000 ng/g were from 90% to 101%. The method gained good repeatability in which RSDs of all levels were less than 16%, and high sensitivity where he LODs ranged between 1.4 and 3.6 ng/g (w/w). For the linear regression equations achieved, the deviation of all back calculated concentrations from true concentrations were lower than 20% with both solvent and matrix-matched calibrations.

### Health risk assessment

Pesticide residue level in a specific vegetable is compared with the maximum residue level (MRL) established by the FAO and WHO (CODEX) [15]. In cases the vegetables are not set up in the CODEX, the respective MRLs regulated by Vietnamese Ministry of Health Circular 50/2016/TT-BYT [32] are used. No comparion was made for pesticides whose MRL were not established.

The estimated daily intake (EDI) was calculated for fenobucarb, fipronil, cypermethrin and difenoconazole in order to identify if any health risk was associated with each pesticide residue in vegetables [33, 34]. Estimated daily intake of pesticide i (EDI_i_) was calculated using the following **Eq (1)**:

$$EDI_{i}=\frac{\sum residue\;level\;of\;pesticide\;i\;in\;vegetable\;j\;(mg/kg)\times daily\;consumption\;of\;vegetable\;j\;(kg/day)}{body\;weight\;(kg)}$$ Eq (1)

Health hazard index of pesticide i (HHI_i_) was then calculated by Eq **(2)**:

$$HHI_{i}=\frac{EDIi}{ADIi}$$ Eq (2)

Where ADIi is the aceptable daily intake of pesticide i.

If HHI_i_ is > 1, then long-term health risk would be associated with the consumption of vegetable j contaminated by pesticide i.

Noticeably, an EDI of a pesticide must be the sum of the residue level of pesticide contained in all sources of food that are consumed. However, this study only focused on four different vegetables mostly consumed at the study sites, therefore, the EDI value is calculated based on the level of pesticide residues in these four studied vegetables.

### Data analysis

#### Statistical analysis of the household survey

Quantitative data collected from the survey (using a structured questionnaire) was analyzed by IBM SPSS Statistics v20 (USA). Descriptive statistics such as mean, median, and frequency were applied to explore the characteristics of pesticide use and farming practices of local farmers.

Qualitative data (collected from in-depth interviews) was kept in notes, categorized, and analyzed accordingly.

#### Statistical analysis of pesticide residues

Sigma Plot version 11.0 (Systat Software Inc, USA) statistic software was used to perform the statistical analysis. Shapiro-Wilk test and Levene test were applied to test the normal distribution of the data (p = 0.05). One-way ANOVA (Kruskal-Wallis ANOVA on Ranks in case of non-normality), or two-sample T-test (Mann-Whitney U Test in case of non-normality) was run to find significant differences between groups.

## Results and discussion

### Pesticide use in vegetable production

The main vegetables cultivated in both sites were mustard greens, green onions, and lettuce (see **S4 Table**). Moreover, in the Quang Tho commune of Thua Thien Hue province, the coast plain, there was a large area of pennywort production of ca. total 8 ha, owned by the local cooperative.

The survey results show that all interviewed households used pesticides during the production cycles (**Table 1**). It is worth noting that 18% of respondents did not remember the names of the most recently used pesticides. There were 24 different active ingredients (4 herbicides, 11 insecticides, 9 fungicides) in 45 registered commercial products (Circular No.10/2019/TT-BNNPTNT [35]) being used in the two survey sites. These figures were comparable with vegetable cultivation in Lam Dong province, which is one of the biggest vegetable suppliers to South Vietnam—(44 commercial products) [36]. Remarkably, seven out of 24 pesticides (29%) were moderately toxic pesticides (class II, WHO classification). Among 18 synthetic pesticides applied, the most frequently used pesticides were acetochlor, pretilachlor, fluazifop-p-butyl, cypermethrin, fenobucarb, fipronil, difenoconazole, isoprothiolane, tebuconazole, trifloxystrobin. These ten were, therefore, considered for further monitoring campaigns on pesticide residues in selected vegetables. Six (in Thua Thien Hue) and five (in Quang Binh) active ingredients were applied at higher doses compared to the instruction on the container labels. For instance, acetochlor, a class III herbicide, was sprayed 1.8–3.5 times higher, and emamectin was used 1.7–3.0 times higher (recorded in both study sites), etc. This situation was similarly found in Lam Dong Province [36] and the Mekong Delta [37].

**Table 1:** Pesticice usage and overdose rate at the study sites.

	Active ingredient	Toxicity class^a^	Usage percentage (% respondent)		Average practical spraying dose (kg/ha)		Recommended dose ^(^^b^^)^ (kg/ha)	Average overdose rate (times)	
			TTH	QB	TTH	QB		TTH	QB
			(n = 155)	(n = 78)	(n = 115)	(n = 69)		(n = 115)	(n = 69)
	**Herbicides**								
1	Acetochlor	III	45	25	0.482	0.252	0.139	3.5	1.8
2	Fluazifop-P-butyl	III	31	42	0.077	0.085	0.150	-	-
3	Pretilachor	U	26	57	0.011	0.125	0.420	-	-
4	Quizalofop-P-Ethyl	NL	8	-	0.005	-	0.006	-	-
	**Insecticides**								
5	Abamectin	NL	35	38	0.001	0.009	0.025	-	-
6	Azadirachtin	NL	68	77	0.001	0.001	0.002	-	-
7	Cypermethrin	II	66	72	0.010	0.0125	0.0125	-	-
8	Emamectin	NL	22	10	0.009	0.005	0.003	3.0	1.7
9	Emamectin- benzoat	NL	84	79	0.018	0.025	0.018	-	1.4
10	Fenobucarb	II	41	33	0.019	0.200	0.280	-	-
11	Fipronil	II	59	43	0.02	0.010	0.025	-	-
12	Flufiprole	NL	68	7	0.06	0.050	0.050	1.2	-
13	Indoxacarb	II	58	8	0.124	0.050	0.100	1.2	-
14	Lufenuron	NL	1	5	0.010	0.020	0.050	-	-
15	Thiamethoxam	NL	22	16	0.005	0.010	0.050	-	-
	**Fungicides**								
16	Carbendazim	U	9	14	0.102	0.222	0.750	-	-
17	Difenoconazole	II	82	39	0.120	0.085	0.062	1.9	1.4
18	Hexaconazole	III	6	-	0.001	-	0.050	-	-
19	Isoprothiolane	II	14	41	0.150	0.120	0.480	-	-
20	Mancozeb	U	28	3	0.469	0.600	2.100	-	-
21	Metalaxyl	II	3	7	0.005	0.010	0.120	-	-
22	Tebuconazole	II	53	66	0.045	0.060	0.060	-	-
23	Trifloxystrobin	U	47	69	0.005	0.020	0.050	-	-
24	Validamycin A	NL	79	90	0.053	0.050	0.025	2.1	2.0

-: no infomation

TTH: Thua Thien Hue province; QB: Quang Binh province

^a^ WHO Classification [38] (Ia extremely hazardous, Ib highly hazardous, II moderately hazardous, III slightly hazardous, U unlike to present acute hazard, NL not listed)

^b^ Recommended dose is based on instruction label on the pesticide container.

Pesticide use and awareness of local farmers in Thua Thien Hue and Quang Binh provinces are summarized in **S5 Table**. The average number of pesticides used for each crop varied from four to six different active ingredients, depending on the stage of the plant and pest situation. This figure was similar to the results published in Hanoi (capital of Vietnam) [14] but lower compared to 9–10 pesticides used in each crop in Vinh Long province (in the Mekong Delta), where farmers tended to change the pesticides after each cropping circle to avoid the pesticide resistance of pests and diseases [37]. In terms of pesticide application technique, 52% of respondents in Thua Thien Hue followed instruction on the containers’ labels. Some (26%), based on personal experience to estimate the doses to be applied. In Quang Binh, many farmers trusted their personal experience (43%) and hardly followed instructions of agricultural extensionists (14% responded). Although there are usually two to three training courses on pesticide use organized by the commune annually, only 32% of respondents in Thua Thien Hue and 22% in Quang Binh admitted to participating regularly. The criteria for pesticide purchasing were mainly "effectiveness for crops" and "cost", which help to increase crop productivity and decrease the price of agricultural products. Only 20% of the respondents were aware of pesticide toxicity. Most of them did not pay attention to the legality of use, the potential environmental risk, or the health risk of pesticides. Not to mention the level of toxicity and classification of pesticides, almost no one has knowledge about this issue. Some farmers did not use any personal protective equipment when handling pesticides. None of the interviewed households had a locked cabinet to store the pesticides. Most respondents (71% and 64% in Thua Thien Hue and Quang Binh, respectively) discarded the empty pesticide containers in the collective trash pin, and about 25% were left in the field. Previous studies, not only in Vietnam [14, 36], but also in other agricultural countries [5, 6, 39], emphasized that this harmful habit is causing environmental damages which will consequently impact human health through interacting with land and water.

### Pesticide residues in vegetables

Based on the survey results, four vegetables in Thua Thien Hue (mustard greens, green onions, lettuce, and pennywort) and three vegetables in Quang Binh (mustard greens, green onions, lettuce) were selected for monitoring the residues of ten most commonly used, synthetic pesticides (acetochlor, pretilachlor, fluazifop-p-butyl, cypermethrin, fenobucarb, fipronil, difenoconazole, isoprothiolane, tebuconazole, and trifloxystrobin).

#### Pollution patterns of total pesticide residues in vegetables

Of all 290 vegetable samples measured, 80.7% contained at least one target pesticide of which 23% were contaminated pesticides that exceeded their MRL values. This figure is higher compared to that reported in the other studies elsewhere such as in Pakistan [40], of which less than 10% of fruit and vegetable samples contained pesticide exceeding their MRLs, in Egypt [41] or Turkey [42] this percentage was 17% (fruit samples). But it was lower than that documented in various studies, such as in Ethiopia [43] (30% vegetable samples contained pesticides above MRLs), or Argentina [44] (56% samples contained pesticides above MRLs). However, it is worth noticed that this comparison is only relative. The proportion of pesticide detections in fruit and vegetables are highly dependent on the number of pesticides in the scope of the analysis methods used in each study. The more pesticides in the scope, the higher the detection frequency. There were 25% of quantified samples in this study recorded with at least four pesticides co-occurred, which might pose a higher risk to human health than the effect caused by individual pesticides [24]. Total pesticide concentrations (sum of individual pesticides in one sample) in the collected samples are summarized in **Table 2**.

**Table 2:** Total pesticide concentrations (mg/kg) and detection frequencies (%) in vegetables collected in Thua Thien Hue and Quang Binh provinces.

Location	Season	Vegetables	Detection frequency (%)	Min—Max (mg/kg)	Median ± MAD ^(^*^)^ (mg/kg)
**Thua Thien Hue Province (n = 162)**	Wet season	Mustard greens (n = 20)	100	< LOD– 0.129	0.052 ± 0.023
		Lettuce (n = 20)	100	< LOD– 0.402	0.083 ± 0.041
		Green onions (n = 22)	100	< LOD– 2.680	0.105 ± 0.075
		Pennywort (n = 10)	100	< LOD– 0.492	0.197 ± 0.125
	Dry season	Mustard greens (n = 30)	80.0	< LOD– 4.836	0.048 ± 0.040
		Lettuce (n = 28)	78.5	< LOD– 0.295	0.014 ± 0.010
		Green onions (n = 22)	81.8	< LOD– 11.934	0.156 ± 0.086
		Pennywort (n = 10)	50.0	< LOD– 1.716	0.041 ± 0.021
	Total	Mustard greens	88.0	< LOD– 4.836	0.050 ± 0.029
		Lettuce	87.5	< LOD– 0.402	0.046 ± 0.042
		Green onions	90.9	< LOD– 11.934	0.118 ± 0.109
		Pennywort	75.0	< LOD– 1.713	0.117 ± 0.117
**Quang Binh Province (n = 128)**	Wet season	Mustard greens (n = 20)	100	< LOD– 1.314	0.038 ± 0.009
		Lettuce (n = 18)	100	< LOD– 8.765	0.110 ± 0.075
		Green onions (n = 20)	95.0	< LOD– 1.373	0.037 ± 0.019
	Dry season	Mustard greens (n = 30)	43.3	< LOD– 38.604	0.055± 0.029
		Lettuce (n = 20)	65.0	< LOD– 0.108	0.034 ± 0.033
		Green onions (n = 20)	50.0	< LOD– 32.117	0.011 ± 0.011
	Total	Mustard greens	66	< LOD– 38.603	0.040 ± 0.018
		Lettuce	81.6	< LOD– 8.765	0.054± 0.043
		Green onions	72.5	< LOD– 32.117	0.030 ± 0.010

^(*)^ MAD—Median absolute deviation

In the wet season, more than 95% of collected samples were contaminated with at least one studied pesticide, while this detection frequency was 80% in the dry season. Particularly, in the case of pennywort in Thua Thien Hue, while 100% of the samples in the wet season were contaminated by the studied pesticides, this figure was only 50% in the dry season. It was explained by the fact that due to being afraid of the rain to wash out pesticides sprayed from plants, farmers tended to apply pesticides more frequently and use shorter pre-harvest intervals than set on the label, causing a high possibility of pesticide residues in vegetables.

Total pesticide residues varied by types of vegetables, by seasons, and by locations. The median total pesticide concentration recorded in Thua Thien Hue fluctuated from 0.014 ± 0.010 mg/kg (lettuce in the dry season) to 0.197 ± 0.125 mg/kg (pennywort in the wet season). Meanwhile, in Quang Binh, the fluctuation was from non-detected (mustard greens in the dry season) to 0.109 ± 0.075 mg/kg (lettuce in the wet season). Maximum total pesticide residue found in mustard greens was up to 38.6 mg/kg (a sample collected in the dry season in Quang Binh), in lettuce was 8.8 mg/kg (wet season in Quang Binh), in green onions was 32.1 mg/kg (dry season in Quang Binh), and in pennywort was 1.7 mg/kg (dry season in Thua Thien Hue). These maximum values were not considered as outliers in the data statistics of this study since they represented the consequence of misuse or indiscrimination of pesticide application at a specific farm. In other words, they would serve as a warning for local authorities to pay more attention to the training and management of pesticide use.

#### Seasonal and spatial variation of target pesticide residues in vegetables

Shapiro-Wilk test demonstrated that the analyzed data were non-normality. Therefore, Mann-Whitney Rank Sum Test and Kruskal-Wallis ANOVA on Ranks were run to identify significant differences in pesticide residues among seasons, vegetables, and provinces. The statistical results are shown in the **S6 Table**.

In terms of seasonal variation, in Thua Thien Hue, pesticide residues in lettuce samples collected in the dry season (median 0.014 ± 0.010 mg/kg, **Table 2**) was significantly lower than that collected in the wet season (median 0.083 ±0.041 mg/kg) with p < 0.001 (**S6 Table**). Similarly, in Quang Binh, total pesticide residues in lettuce (median 0.034 ± 0.033 mg/kg) in the dry season was significantly lower compared to those in the wet season (0.110 ± 0.075 mg/kg with p < 0.001 (**Table 2, S6 Table**).

Regarding the differences between vegetables, in Thua Thien Hue, ANOVA results revealed the significant higher residues of pesticides in green onions (0.118 ± 0.109 mg/kg) compared to that of lettuce (0.046 ± 0.042 mg/kg) and mustard greens (0.050 ± 0.029 mg/kg) with p = 0.003 and p = 0.02, respectively (**Table 2, S6 Table)**. The explanation for these differences could be due to the habit of pesticide spraying of green onion farmers in Thua Thien Hue, who traditionally applied pesticides ca. 5 times per crop (especially, there was a case applying pesticide every week (8 times per crop), regardless the presence of pests/diseases or not, *household interview data*) with high dosage, contemporaneous shortening the pre-harvest interval. This implied a potential health risk of pesticide intake to green onion consumers in Thua Thien Hue province. Meanwhile, in Quang Binh province, pesticide residue levels were not different among vegetables (p > 0.05) (**S6 Table**).

When comparing pesticide residues in vegetables collected at the two provinces, it turned out that mustard greens and green onions collected in Thua Thien Hue (0.050 ± 0.029 mg/kg and 0.118 ± 0.109 mg/kg, respectively) were significantly more contaminated than those in Quang Binh (0.031 ± 0.021 mg/kg and 0.030 ± 0.010 mg/kg, respectively) (p < 0.05) (**Table 2, S6 Table**). This finding may be linked to the facts that local farmers in Quang Binh used fewer pesticides per crop (average 4 pesticides) and lower spraying frequency (average 3 times) compared to ones in Thua Thien Hue (5 pesticides and 4.5 times, respectively) (**Table 1**).

#### Occurrence of individual pesticides in vegetables

The results of single pesticide residues in vegetables are shown in **Table 3**

**Table 3:** Concentration (mg/kg), detection frequency (%), and frequency of samples that contained pesticide exceeding the MRL (%) of each studied pesticide in vegetables collected in Thua Thien Hue and Quang Binh provinces.

Pesticides	TT Hue (n = 162)				Quang Binh Province (n = 128)				Total (n = 290)			
	Detection frequency (%)	Median ± MAD ^(^^a^^)^ (mg/kg)	Min—Max (mg/kg)	Samples > MRL (%) ^(b)^	Detection frequency (%)	Median ± MAD ^(^^a^^)^ (mg/kg)	Min—Max (mg/kg)	Samples > MRL (%) ^(b)^	Detection frequency (%)	Median ± MAD ^(^^a^^)^ (mg/kg)	Min—Max (mg/kg)	Samples > MRL (%) ^(^^b^^)^
Fenobucarb	36.4	0.004 ± 0.001	< LOD– 0.141	6.2	36.7	0.011 ± 0.004	< LOD -0.083	22.7	36.6	0.008 ± 0.005	< LOD– 0.141	13.4
Acetocrhlo	11.7	0.004 ± 0.001	< LOD– 1.716	2.5	14.1	0.022 ± 0.013	< LOD– 0.653	11.7	12.8	0.010 ± 0.006	< LOD– 1.716	6.6
Fipronil	42.6	0.023 ± 0.018	< LOD– 3.710	24.1	10.2	0.009 ± 0.006	< LOD– 1.295	2.3	28.3	0.021 ± 0.017	< LOD– 3.710	14.5
Pretilachlor	12.3	0.017 ± 0.012	< LOD– 0.110	8.6	17.2	0.019 ± 0.009	< LOD– 1.013	14.1	14.5	0.017 ± 0.008	< LOD– 1.013	11.0
Fluzifop-p-butyl	12.3	0.006 ± 0.003	< LOD– 0.054	3.1	2.3	0.007 ±0.0008	< LOD– 0.008	0.0	7.9	0.007 ± 0.003	< LOD– 0.054	1.7
Isoprothiolane	6.2	0.010 ± 0.006	< LOD– 0.029	3.1	4.7	0.008 ± 0.005	< LOD– 0.491	2.3	5.5	0.010 ± 0.007	< LOD– 0.491	2.8
Trifloxystrobin	21	0.007 ± 0.004	< LOD– 0.593	1.9	0	-	-	0	11.7	0.007 ± 0.004	< LOD– 0.593	1.0
Tebbuconazole	22.2	0.019 ± 0.015	< LOD– 3.594	0.6	2.3	0.012 ± 0.002	< LOD– 0.013	0	13.4	0.014 ± 0.010	< LOD—3.594	0.3
Cypermethrin	79.6	0.040 ± 0.024	< LOD– 11.860	7.4	62.5	0.033 ± 0.021	< LOD– 30.295	6.3	72.1	0.037 ± 0.024	< LOD– 30.295	6.9
Difenoconazole	60.5	0.011± 0.006	< LOD– 4.371	0.6	23.4	0.020 ± 0.014	< LOD– 15.443	0.8	44.1	0.017 ± 0.007	< LOD– 15.443	0.7

(a) MAD—Median absolute deviation

(b) Percentage of samples that contained pesticide exceeding the MRL

In general, cypermethrin was detected with the highest frequency (72.1% of all analyzed samples) as well as the highest median concentration (0.037 ±0.0 24 mg/kg), followed by difenoconazole (44.1%). This is in line with the survey findings (**Table 1**), which discovered cypermethrin and difenoconazole were the most applied insecticides and fungicides, respectively, in the two study sites. Remarkably, less than 7% of vegetable samples containing cypermethrin and difenoconazole exceeded their MRL values. Anyhow, these two pesticides are categorized as moderately hazardous (class II). Some studies also reported the considerable residues of cypermethrin and difenoconazole in fruit or vegetable samples [20, 34, 40, 45, 46],. Meanwhile, isoprothiolane, even though was used at the study sites, was quantified at the lowest frequency (5.5% of total samples), and exceeded its MRL value in 3% of samples. This finding is similar with the report from Korea where isoprothiolane was found in only one sample and its content also exceed the respective MRL [47]. Fluazifop-p-butyl and trifloxystrobin occurred at the lowest detectable concentrations of all the study pesticides (0.007 ± 0.003 and 0.007 ± 0.004 mg/kg, respectively), and exceeded the MRL in less than 2% of analyzed samples, although they were used at the considerable frequency at both study sites (> 30% of interviewed farmers admitted, **Table 1**). Their rapid decomposition (**S1 Table**) helps reduce the residue levels in the food matrix.

Regarding the vegetables taken in Thua Thien Hue province, up to 79.6% and 60.5% of analyzed samples were contaminated by cypermenthrin and difenoconazole, respectively, while only 6.2% and 11.7% of samples contained a detectable amount of isoprothiolane and acetochlor. Median quantified concentrations of the target pesticides varied from 0.004 ± 0.001 mg/kg, max 0.141 mg/kg (fenobucarb) to 0.040 ± 0.024 mg/kg, max 11.9 mg/kg (cypermethrin). The pesticide that was found to most frequently exceed its MRL value was fipronil (39 out of 162 analyzed samples). The MRL exceedance proportion of other pesticides was all less than 10%.

In Quang Binh province, a slightly different pattern was recorded. Cypermethrin was detected at the highest frequency (62.5% samples), and the median residue level was significantly high (0.033 ± 0.021 mg/kg, with 8 samples exceeding the MRL value). Trifloxystrobin was not found in any vegetable samples, and tebuconazole and flozifop-p-butyl occurred in only of 2.3% analyzed samples. Among the ten studied pesticides, febobucarb was most frequently exceeded its MRL value (in 22.7% samples), followed by pretilachlor (14.1%) then acetochlor (11.7%).

Mann-Whitney Rank Sum Test was run to identify the possibility of seasonal or spatial impacts on the residue levels of individual pesticides. However, no significant differences were found.

One remarkable thing in this study which is worth mentioning is that, unlike most of the other countries where cauliflower, broccoli, asparagus, carrot, celery, cucumber, etc., are frequently consumed, Vietnamese people prefer leafy vegetables, such as mustard greens, lettuce, sweet potato leaves or green onions, as the main source for vitamins and fiber supplements. Some of them do not exist in the commodity list in the Codex Pesticides Residues in Food Online Database [16]. This lack of information has caused some disadvantages when comparing the findings of this study with the others. Therefore, it is recommended for FAO and WHO to conduct more investigation on leafy vegetables, such as the ones in this study, to set up more relevant MRL data.

### Health risk assessment

An ADI is set up based on "data on the biochemical, metabolic, pharmacological, and toxicological properties of the pesticide derived from studies of experimental animals and observations in humans" [48] and "calculated by dividing the overall no-observable-effect level (NOEL) from the animal studies by a safety factor" [49]. To have an appropriate Health hazard index (HHI) value for each pesticide, only the pesticide with detection frequency higher than 30% were taken into account as contributors to the EDI calculation [46]. As a result, only fenobucarb, fipronil, cypermethrin, and difenoconazole detected in vegetables in Thua Thien Hue, and fenobucarb and cypermethrin residues in vegetables in Quang Binh, were adequate for calculating the related EDI. Moreover, this study applied a worst-case scenario, assuming that, in general, interviewed people consumed simultaneously all studied vegetables, and pesticides were not washed out or decomposed during preparation for a meal. In other words, these are the maximum calculated EDIs.

Based on the survey findings, the average body weight of vegetable consumers and the mean daily consumption of each vegetable were discovered. Accordingly, EDI values (**Eq 1**) and health hazard index (**Eq 2**) for the potential pesticides (fenobucarb, fipronil, cypermethrin, and difenoconazole) were calculated and shown in **Table 4.**

**Table 4:** Health risk of pesticide residues in vegetables collected in Thua Thien Hue and Quang Binh provinces.

		Hue (n = 155)				Quang Binh (n = 78)			ADI (mg/kg/day)	EDIs (mg/kg bw/day)		HHIs	
	Body weight ± SD (kg)	63.3 ± 12.3				64.9 ± 8.0				Hue	Quang Binh	Hue	Quang Binh
	Consumption ± SD (kg/day)	Mustard	Lettuce	Onions	Pennywort	Mustard	Lettuce	Onions					
		0.167	0.106	0.034	0.090	0.211	0.107	0.310					
		± 0.072	± 0.066	± 0.019	± 0.111	± 0.769	± 0.612	± 0.178					
Average concentration (mg/kg)	Fenobucarb	0.005	0.002	0.014	0.037	0.010	0.037	0.015	-	8.10^−5^	10.10^−5^	-	-
	Acetochlor	-	-	-	-	-	-	-	360.10^−5^	-	-	-	-
	Fipronil	0.048	0.017	0.063	0.196	-	-	-	20.10^−5^	46.10^−5^	-	2.32	**-**
	Pretilachlor	-	-	-	-	-	-	-	0.018	-	-	-	-
	Fluzifop-p-butyl	-	-	-	-	-	-	-	0.10	-	-	-	-
	Isoprothiolane	-	-	-	-	-	-	-	0.10	-	-	-	-
	Trifloxystrobin	-	-	-	-	-	-	-	0.03	-	-	-	-
	Tebbuconazole	-	-	-	-	-	-	-	0.03	-	-	-	-
	Cypermethrin	0.201	0.066	0.996	0.107	0.880	0.549	1.772	0.05	131.10^−5^	462.10^−5^	0.03	0.09
	Difenoconazole	0.037	0.010	0.233	0.088	-	-	-	0.01	36.10^−5^	-	0.036	-

Regarding vegetables cultivated in Thua Thien Hue province, EDI values ranged from 8.10^−5^ mg/kg bw/day (fenobucarb) to 131.10^−5^ mg/kg bw/day (cypermethrin). In Quang Binh, EDI of fenobucarb was 10.10^−5^ mg/kg bw/day and that of cypermethrin was 462.10^−5^ mg/kg bw/day. So far, there has been no ADI value for fenobucarb, therefore its HHI could not be estimated.

Among the studied pesticides, health risk was related to the residues of fipronil in vegetables collected in Thua Thien Hue province (EDI was 46.10^−5^ mg/kg bw/day) with HHI values of 2.32, proposing a warning alarm for vegetable consumers in this region. Other studies elsewhere, such as in China [50] or Belgium [51], also documented the potential risk of fipronil in food stuff. Fipronil is a phenyl-pyrazole insecticide, categorized as a moderately hazardous compound (class II, [38]), and has systemic nature which helps it to be absorbed into the plant and translocate in the tissues [52]. The high toxicity and its systemic property could be two of the reasons that its ADI is very low (20.10^−5^ mg/kg).

The considerably intensive spraying schedule in Thua Thien Hue Province (average 4.5 times per crop cycle, **S5 Table**), in association with lack of knowledge about pesticide toxicity among local farmers might result in the high detection frequency of not only fipronil but other pesticides such as cypermethrin, difenoconazole, or fenobucarb in samples. To reduce the possibility of health risks associated with pesticide residues in vegetables, consumers are recommended to prepare carefully the vegetables before eating. For example, washing with clean water or cooking well, which has been published as an effective tool for pesticide removal from the vegetable surface [34, 53].

## Conclusions

This study recorded the widespread use of pesticides in vegetable production by farmers and the occurrence of ten pesticides in vegetables collected from the two provinces in Central Vietnam. Farmers tended to over-rely on personal experience when purchasing pesticides while lacking knowledge on pesticide toxicity. Most of the collected vegetable samples contained pesticides in varying degrees. Some had pesticide residues above the MRL values. Cypermethrin, difenoconazole, fipronil, and fenobucarb were found at high frequency. Wet season samples exhibited higher pesticide residues in comparison with dry season samples. This study also discovered a health risk associated with fipronil residues in vegetables in Thua Thien Hue Province.

Therefore, to protect farmers and consumers and to ensure sustainability of agricultural sector, some following recommendations need to be considered: i) Training sessions should be organized with an easy-to-understand method to educate farmers on the consequences of misusing pesticides. These require an approach that facilitates the exchange and cooperation among stakeholders (farmers, authorities, retailers, and producers); ii) Safe pesticide usage should be promoted, such as implementing effective crop protection regulations and raising awareness about pesticide residues in various agricultural products; iii) Monitoring campaigns should be expanded to various sources: farms, markets, including organic food stores, imported foods, etc.

## Supporting information

S1 Text Analytical procedure for quantification of pesticides in vegetables. (DOCX)

S1 Table General properties^a^ of the target pesticides. (DOCX)

S2 Table Spike recovery and precision of the developed method at five fortified levels prepared in mustard green and green onion matrixes. (DOCX)

S3 Table Linear regression equations of solvent and matrix-matched calibrations. (DOCX)

S4 Table Main vegetables cultivated at the two study sites. (DOCX)

S5 Table Pesticide use and awareness of local farmers in Thua Thien Hue and Quang Binh Provinces. (DOCX)

S6 Table Comparison on pesticide residues in vegetables of Thua Thien Hue and Quang Binh Provinces. (DOCX)
