# Supplementary material for: Assessment of pesticide use and pesticide residues in vegetables from two provinces in Central Vietnam
Source: PLoS One. 2022 Jun 13;17(6):e0269789. doi: 10.1371/journal.pone.0269789 (PMC9191740; doi:10.1371/journal.pone.0269789)
Supplement: S1 Text — (DOCX) [file pone.0269789.s001.docx]

**S1 Text. Analytical procedure for quantification of pesticides in vegetables [29]:**

Five grams of vegetable sample were spiked with δ-HCH (100 ng) acting as surrogate. Acetone (60 mL) were used for ultrasonic extraction in 15 min and twice replicated with 20 mL acetone each time. Clean-up steps comprised activated carbon-packed column to eliminate pigments and non-polar compounds from the matrix, followed by florisil cartridges to remove polar compounds. The extract was evaporated to nearly dryness. After ca. one mL of toluene was added, the sample was transferred to an amber vial containing 100 ng phenanthrene-D10. The vial was stored at -20 ^o^C until GC/MS/MS measurement.

Gas chromatography separation employed a gas chromatography - triple quadrupole mass spectrometry system (GC-MSTQ model 8040, Shimadzu, Japan), Rtx-CL pesticide capillary column (30 m x 0.25 mm, film thickness 0.25 µm, Restek, USA). Temperature program was used as follows: Initial temperature 70 ^o^C, kept for 1 min, increased by 10 ^o^C/min to 180 ^o^C, then by 5 ^o^C/min^1^ to 195 ^o^C, and by 8 °C to 219 ^o^C, kept for 5 min, then increased further by 3 ^o^C/min to 240 ^o^C, and finally by 10 ^o^C min^-1^ to 280 ^o^C, held for 6 min. Interface temperature of the mass spectrometer was 250 ^o^C, ion source temperature was 200 ^o^C, and detector voltage was 1.5 kV. Multiple reaction monitoring (MRM) was applied. The NIST-11 database V2.0 (NIST/EPA/NIH Mass Spectral Database) was used for pesticide qualification and m/z identification. Internal standard calibration was used for quantitative analysis.
