# Supplementary material for: Assessment of pesticide use and pesticide residues in vegetables from two provinces in Central Vietnam
Source: PLoS One. 2022 Jun 13;17(6):e0269789. doi: 10.1371/journal.pone.0269789 (PMC9191740; doi:10.1371/journal.pone.0269789)
Supplement: S1 Table — (DOCX) [file pone.0269789.s002.docx]

**S1 Table. General properties^a^ of the target pesticides**

| **Compounds** | **Class** | **Mode of action** | **Solubility** | **Vapor pressure (20^o^C)** |  | **Octanol/water partition coefficient** | **Soil sorption (K_oc_**) | **^*^WHO toxicity class** |
| --- | --- | --- | --- | --- | --- | --- | --- | --- |
|  |  |  | **(20^o^C)** |  | **Half-life in soil** | **(LogK_ow_)** **(pH 7, 20^o^C)** |  |  |
|  |  |  | mg/L | mPa | days |  | L/kg |  |
| **Herbicides** |  |  |  |  |  |  |  |  |
| Acetochlor | Chloroacetamide | Selective | 282 | 0.022 | 12.1 | 4.14 | 156 | III |
| Pretilachlor | Chloroacetamide | Selective | 500 | 0.133 | 30 | 4.08 | - | U |
| Fluazifop-P-butyl | Aryloxyphenoxy-  propionate | Selective | 0.93 | 0.12 | 8.2 | 4.5 | 3394 | III |
| **Fungicides** |  |  |  |  |  |  |  |  |
| Tebuconazole | Azole | Systemic | 32 | 0.0013 | 62 | 3.7 | - | III |
| Difenoconazole | Azole | Systemic | 15 | 3.33 10^-5^ | 130 | 4.36 | - | II |
| Isoprothiolane | Dithiolane | Systemic | 54 | 18.8 | - | 3.3 | 1352 | II |
| Trifloxystrobin | Strobin | Broad spectrum | 0.61 | 0.0034 | 7 | 4.5 | 6700 | III |
| **Insecticides** |  |  |  |  |  |  |  |  |
| Fenobucarb | Carbamate | Contact acting | 420 | 48 | 18.5 | 2.78 | 1068 | II |
| Fipronil | Pyrazole | Broad spectrum | 3.78 | 0.002 | 142 | 3.75 | - | II |
| Cypermethrin | Pyrethroid | Non-systemic | 0.009 | 0.0068 | 60 | 5.55 | 350000 | II |

^a^ <http://sitem.herts.ac.uk/aeru/ppdb/en/atoz.htm>

^*^ WHO Classification [38] (Ia extremely hazardous, Ib highly hazardous, II moderately hazardous, III slightly hazardous, U unlike to present acute hazard, NL not listed)
