# Supplementary material for: Assessment of pesticide use and pesticide residues in vegetables from two provinces in Central Vietnam
Source: PLoS One. 2022 Jun 13;17(6):e0269789. doi: 10.1371/journal.pone.0269789 (PMC9191740; doi:10.1371/journal.pone.0269789)
Supplement: S2 Table — (DOCX) [file pone.0269789.s003.docx]

**S2 Table. Spike recovery and precision of the developed method at five fortified levels prepared in mustard green and green onion matrixes**

| **Compounds** | **LOD** | **Mustard greens (n = 5)** | | | | | | | | | | **Green onions (n = 5)** | | | | | | | | | |
| --- | --- | --- | --- | --- | --- | --- | --- | --- | --- | --- | --- | --- | --- | --- | --- | --- | --- | --- | --- | --- | --- |
|  | **(ng/g) (n=7)** | Fortified | | Fortified | | Fortified | | Fortified | | Fortified | | Fortified | | Fortified | | Fortified | | Fortified | | Fortified | |
|  |  | 5 ng/g | | 20 ng/g | | 100 ng/g | | 500 ng/g | | 2000 ng/g | | 5 ng/g | | 20 ng/g | | 100 ng/g | | 500 ng/g | | 2000 ng/g | |
|  |  | Rev^a^ | RSD | Rev^a^ | RSD | Rev^a^ | RSD | Rev^a^ | RSD | Rev^a^ | RSD | Rev^a^ | RSD | Rev^a^ | RSD | Rev^a^ | RSD | Rev^a^ | RSD | Rev^a^ | RSD |
|  |  | (%) | (%) | (%) | (%) | (%) | (%) | (%) | (%) | (%) | (%) | (%) | (%) | (%) | (%) | (%) | (%) | (%) | (%) | (%) | (%) |
| Fenobucarb | 2 | 79 | 13 | 91 | 9 | 103 | 2 | 97 | 5 | 95 | 4 | 78 | 9 | 92 | 9 | 103 | 4 | 97 | 4 | 90 | 4 |
| δ-HCH ^b^ | 1.4 | 107 | 12 | 93 | 10 | 89 | 4 | 99 | 3 | 101 | 3 | 90 | 9 | 84 | 8 | 93 | 6 | 97 | 3 | 101 | 2 |
| Acetochlor | 2.6 | 79 | 11 | 103 | 7 | 84 | 6 | 90 | 3 | 94 | 3 | 86 | 13 | 90 | 11 | 89 | 8 | 92 | 4 | 92 | 4 |
| Fipronil | 1.8 | 83 | 7 | 111 | 8 | 91 | 4 | 93 | 4 | 99 | 3 | 88 | 15 | 100 | 10 | 98 | 7 | 95 | 4 | 98 | 3 |
| Pretilachlor | 2.1 | 80 | 12 | 97 | 6 | 87 | 4 | 98 | 4 | 94 | 3 | 98 | 14 | 90 | 8 | 90 | 7 | 89 | 4 | 102 | 3 |
| Isoprothiolane | 2.8 | 79 | 14 | 89 | 10 | 92 | 6 | 96 | 4 | 98 | 4 | 81 | 14 | 88 | 8 | 83 | 3 | 89 | 4 | 91 | 5 |
| Fluazifop-p-butyl | 2.4 | 85 | 12 | 86 | 9 | 98 | 4 | 95 | 4 | 91 | 2 | 82 | 15 | 89 | 7 | 84 | 5 | 91 | 4 | 97 | 3 |
| Trifloxystrobin | 2.4 | 82 | 10 | 86 | 8 | 90 | 2 | 90 | 3 | 90 | 3 | 90 | 12 | 88 | 8 | 99 | 7 | 94 | 4 | 99 | 4 |
| Terbuconazole | 2.2 | 88 | 11 | 84 | 10 | 82 | 3 | 90 | 4 | 96 | 2 | 80 | 7 | 83 | 8 | 86 | 5 | 89 | 4 | 92 | 2 |
| Cypermethrin | 3.6 | 76 | 14 | 87 | 9 | 83 | 4 | 88 | 5 | 91 | 4 | 73 | 12 | 85 | 6 | 86 | 5 | 92 | 5 | 90 | 5 |
| Difenoconazole | 3.1 | 77 | 12 | 83 | 8 | 81 | 5 | 90 | 6 | 90 | 4 | 93 | 16 | 87 | 7 | 94 | 4 | 90 | 5 | 91 | 5 |
