# Supplementary material for: Assessment of pesticide use and pesticide residues in vegetables from two provinces in Central Vietnam
Source: PLoS One. 2022 Jun 13;17(6):e0269789. doi: 10.1371/journal.pone.0269789 (PMC9191740; doi:10.1371/journal.pone.0269789)
Supplement: S3 Table — (DOCX) [file pone.0269789.s004.docx]

**S3 Table. Linear regression equations of solvent and matrix-matched calibrations**

| **Compounds** |  | **Solvent calibration**  Linear regression equations^*^; (Dev %) ^**^ | **Matrix-matched calibration**  Linear regression equations^*^; (Dev %)^**^ |
| --- | --- | --- | --- |
| Fenobucarb |  | y = 0.0129x - 0.1291; (1 – 4) | y = 0.0064x - 0.0167; (1 - 20) |
| δ-HCH^***^ |  | y = 0.0067x - 0.017; (1 – 9) | y = 0.0045x + 0.0352; (1 – 18) |
| Acetochlor |  | y = 0.0326x - 0.4834; (0 – 7) | y = 0.0037x - 0.0025; (1 – 20) |
| Fipronil |  | y = 0.0588x - 1.3988; (1 – 9) | y = 0.0021x - 0.0061; (1 – 19) |
| Pretilachlor |  | y = 0.1595x - 3.0485; (1 – 7) | y = 0.0025x - 0.0108; (0 – 14) |
| Isoprothiolane |  | y = 0.0308x - 0.5577; (1 – 6) | y = 0.0048x - 0.0227; (9 – 16) |
| Fluazifop-p-butyl |  | y = 0.1258x - 2.45; ( 1 – 7) | y = 0.0081x - 0.03; (1 – 18) |
| Trifloxystrobin |  | y = 0.0643x - 1.3808; (1 – 6) | y = 0.0057x + 0.0187; (1 – 10) |
| Tebuconazole |  | y = 0.036x - 0.6514; (1 – 8) | y = 0.0025x - 0.0039; (1 – 20) |
| Cypermethrin |  | y = 0.0413x - 0.8176; (1 – 7) | y = 0.0042x + 0.2533; ( 1 -18) |
| Difenoconazole |  | y = 0.0662x - 1.2508; (1 – 6) | y = 0.002x - 0.007; (2 – 20) |

** In the equations, x is the pesticide concentration, y is the ratio between peak area of analyte and peak area of IS*

*** deviation of back calculated concentration from true concentration (%)*

**** Surrogate*
