# Supplementary material for: Assessment of pesticide use and pesticide residues in vegetables from two provinces in Central Vietnam
Source: PLoS One. 2022 Jun 13;17(6):e0269789. doi: 10.1371/journal.pone.0269789 (PMC9191740; doi:10.1371/journal.pone.0269789)
Supplement: S4 Table — (DOCX) [file pone.0269789.s005.docx]

**S4 Table. Main vegetables cultivated at the two study sites**

|  | **Commodity** | **Percentage of households cultivated (%)** | |
| --- | --- | --- | --- |
|  |  | Thua Thien Hue  (n = 155) | Quang Binh  (n = 78) |
|  |  |  |  |
| 1 | Mustard greens | 65 | 71 |
| 2 | Lettuce | 76 | 52 |
| 3 | Green onions | 48 | 58 |
| 4 | Cilantro | 6 | 10 |
| 5 | Pennywort | 22 | 12 |
| 5 | Sweet potatos | 11 | 18 |
| 6 | Celery | 15 | 7 |
| 7 | Basella alba | 21 | 10 |
| 8 | Amaranth | 20 | 21 |
| 9 | *Glebionis coronaria* | 29 | 9 |
