# Supplementary material for: Assessment of pesticide use and pesticide residues in vegetables from two provinces in Central Vietnam
Source: PLoS One. 2022 Jun 13;17(6):e0269789. doi: 10.1371/journal.pone.0269789 (PMC9191740; doi:10.1371/journal.pone.0269789)
Supplement: S5 Table — (DOCX) [file pone.0269789.s006.docx]

**S5 Table. Pesticide use and awareness of local farmers in Thua Thien Hue and Quang Binh Provinces**

| **Farming practices and awareness** | | **Options** | **Respondent** | |
| --- | --- | --- | --- | --- |
|  |  |  | **TTH (n = 155)** | **QB (n = 78)** |
| 1 | Average farm size (ha) |  | 0.083 | 0.076 |
| 2 | Pesticide spraying frequency (times per crop) |  | 4.5 | 3 |
| 3 | Number of pesticides used per crop per farm |  | 6 | 4 |
| 4 | Experience of using pesticides (%) | Personal experience | 26 | 43 |
|  |  | Extensionists | 13 | 14 |
|  |  | Instruction on container label | 52 | 21 |
|  |  | Retailers | 9 | 22 |
| 5 | Criteria of pesticide selection (%) | Price | 23 | 14 |
|  |  | Efficiency | 49 | 61 |
|  |  | Low toxicity | 21 | 19 |
|  |  | Legal to use | 7 | 6 |
| 6 | Knowledge on pesticide side effects (%) | Skin | 68 | 90 |
|  |  | Eyes | 21 | 8 |
|  |  | Respiratory | 43 | 33 |
|  |  | Cancer | 12 | 29 |
|  |  | No effects | 11 | 4 |
| 7 | Personal protective equipment (%) | Yes, always | 59 | 72 |
|  |  | Yes, sometimes | 28 | 23 |
|  |  | Never | 13 | 5 |
| 8 | Storage (%) | Separated storehouse | 32 | 46 |
|  |  | Corner of the house | 30 | 19 |
|  |  | On the field | 38 | 35 |
| 9 | Disposal (%) | Collective trash pit | 71 | 64 |
|  |  | Burning | 7 | 3 |
|  |  | On the field | 21 | 33 |
|  |  | Into canals | 1 | 0 |
| 10 | Participate in technical training courses on pesticide use (%) | Regularly | 32 | 22 |
|  |  | Sometimes | 36 | 53 |
|  |  | Rarely | 19 | 17 |
|  |  | Never | 13 | 8 |
