# Supplementary material for: Assessment of pesticide use and pesticide residues in vegetables from two provinces in Central Vietnam
Source: PLoS One. 2022 Jun 13;17(6):e0269789. doi: 10.1371/journal.pone.0269789 (PMC9191740; doi:10.1371/journal.pone.0269789)
Supplement: S6 Table — (DOCX) [file pone.0269789.s007.docx]

**S6 Table. Comparison on pesticide residues in vegetables of Thua Thien Hue and Quang Binh Provinces**

|  | **Factor** |  | **p-value** | **Significance** |
| --- | --- | --- | --- | --- |
| **Thua Thien Hue Province** | **Season** | Mustard greens | 0.656 | No |
|  |  | Lettuce | <0.001 | Yes |
|  |  | Green onions | 0.916 | No |
|  |  | Penny wort | 0.087 | No |
|  | **Vegetables** | Green onions - Mustard | 0.022 | Yes |
|  |  | Green onions - Lettuce | 0.003 | Yes |
|  |  | Green onions - Pennywort | 0.357 | No |
|  |  | Mustard - Lettuce | 0.677 | No |
|  |  | Mustard - Pennywort | 0.099 | No |
|  |  | Lettuce - Pennywort | 0.055 | No |
| **Quang Binh Province** | **Season** | Mustard greens | 0.075 | No |
|  |  | Lettuce | <0.001 | Yes |
|  |  | Green onions | 0.404 | No |
|  | **Vegetables** | Green onions - Mustard | 0.51 | No |
|  |  | Green onions - Lettuce | 0.291 | No |
|  |  | Mustard - Lettuce | 0.05 | No |
| **Provinces** | | Mustard greens | 0.029 | Yes |
|  |  | Lettuce | 0.705 | No |
|  |  | Green onions | 0.007 | Yes |
